# Progress report on the MindView brain PET detector module based on large area SiPMs arrays

**DOI:** 10.1186/2197-7364-1-S1-A66

**Published:** 2014-07-29

**Authors:** Antonio J González, Stan Majewski, Pablo Conde, Liczandro Hernández, Filomeno Sánchez, Pablo Bellido, Efrén Crespo, Amadeo Iborra, Laura Moliner, Juan P Rigla, Maria J Rodríguez-Álvarez, Michael Seimetz, Antonio Soriano, Alexander Stolin, Luis F Vidal, José M Benlloch

**Affiliations:** Institute for Instrumentation in Molecular Imaging, CSIC-UPV, Valencia, Spain; Center for Advanced Imaging, West Virginia University, Morgantown, WV USA

The main technical goal of the MindView project and, thus, the subject of this contribution, is achieving simultaneous PET/MR imaging, dedicated to brain examination in clinical research settings to study psychiatric disorders. The system will allow visualization of the relevant structures in the brain with very high resolution (~1mm) and sensitivity.

This brain PET will be mounted together with a bird-cage type TR RF-coil. An effective combined PET/RF aperture of 27 cms will be generated. Preliminary detector head tests have been carried out with standard arrays of 12x12 SiPMs of the type FB-30035 from SensL. Several crystal configurations, including small size crystal arrays but also thick monolithic slabs, have been tried with the goal to optimize the best trade-off detector performance. The chosen readout electronics is based on a diode network capable to return independent information about each matrix row and column signals. Therefore, up to 24 outputs can be currently considered for the photon impact reconstruction within the crystal.

In the pilot test with the 10 mm thick LYSO crystal array of ~1.5 mm pitch pixels covering the entire 5x5 cm^2^ photosensor area are seen very well resolved, including the corners. Energy resolutions of individual crystal pixels as good as 10 % are achieved. The detector assembly was typically run at a stable environment temperature of 10-15 Celsius and at 29.5 ± 1.0 Vop bias voltage (~5 V overvoltage). Additionally to these tests, it has also been shown that the system has capability to resolve two staggered layers of 1.5 mm pixel size crystals. The study continues with more layers in the stack.

The MRI compatibility of the SensL SiPMs was shown in during the tests in a 3 Tesla MRI @WVU. Development of MRI compatible circuitry (printed circuits and connectors, etc) is in progress.

